# Abdominal infection plays a role in the incidence of ventilator-associated pneumonia

**DOI:** 10.1186/cc11984

**Published:** 2013-03-19

**Authors:** GB Bouroche, S Ruckly, B Misset, JF Timsit, F Philippart

**Affiliations:** 1 Institut Gustave Roussy, Villejuif, France; 2 Institut Albert Bonniot, La Tronche, France; 3 Hôpital Saint Joseph, Paris, France; 4 CHU Grenoble, France

## Introduction

Despite many therapeutic interventions, ventilator-acquired pneumonias (VAP) are frequent in the ICU and are associated with major morbidity and mortality. Sepsis causes a time-dependent modification of the inflammatory response. This reprogramming could promote the occurrence of a secondary infection and worsen the prognosis. In animals, peritonitis is associated with an alteration of pulmonary immunity and an increasing mortality from secondary pneumonia.

## Methods

To investigate, in humans, the potential involvement of previous intra-abdominal infection (IIA) in preventing or promoting VAP, we realized a prospective observational study using data from a multicenter database (OUTCOMEREA), including all patients admitted to the ICU for severe sepsis or septic shock who required mechanical ventilation for at least 72 hours.

## Results

**Figure 1:**
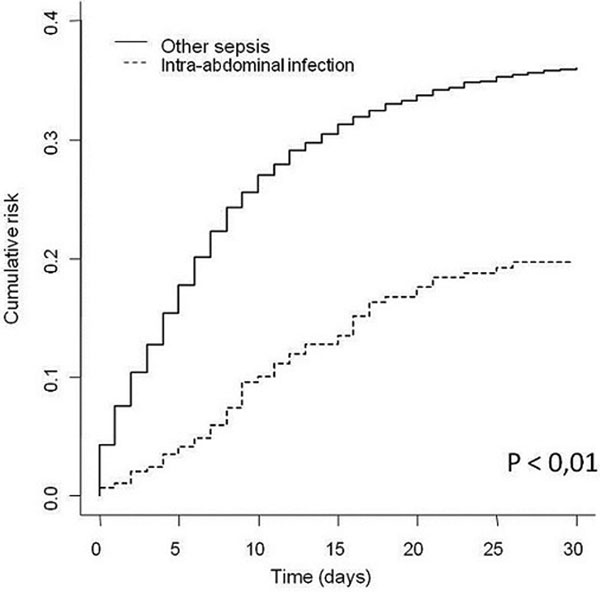
**Cumulative incidence of VAP**.

**Figure 2:**
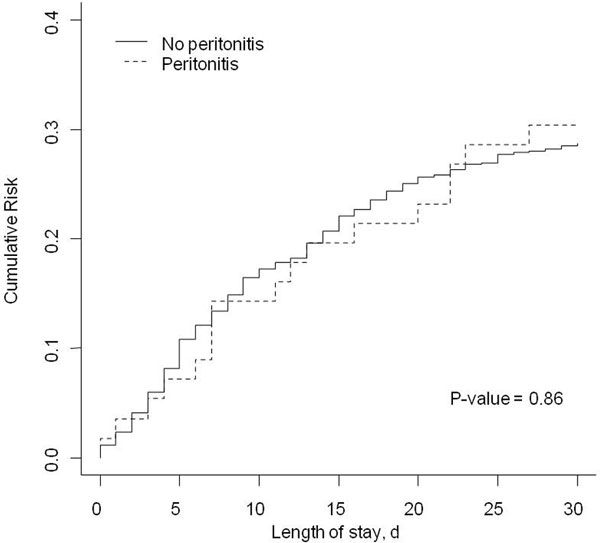
**Cumulative occurrence of death**.

In total, 2,623 patients were included, of which 290 had an IIA. A total of 862 patients (33%) developed a VAP, 56 (19%) in the IIA group and 806 (34%) in the non-IIA group (*P *< 0.01). VAP, after sepsis, occurred less frequently and later in patients with IIA. The occurrence of IIA, in comparison with another sepsis, is a protective factor against VAP (HR = 0.643 (0.478 to 0.863), *P *= 0.003). There is, however, no significant difference between the groups in terms of ICU mortality (28% vs. 32%, *P *= 0.16). See Figures 1 and 2.

## Conclusion

In this study, the presence of an abdominal sepsis, in a context of severe sepsis or septic shock, was associated with a lower incidence of later VAP. These results have to be confirmed in other studies, especially prospective. They open interesting new research directions.

